# Development of *Staphylococcus aureus* tolerance to antimicrobial photodynamic inactivation and antimicrobial blue light upon sub-lethal treatment

**DOI:** 10.1038/s41598-019-45962-x

**Published:** 2019-07-01

**Authors:** Aleksandra Rapacka-Zdonczyk, Agata Wozniak, Michal Pieranski, Anna Woziwodzka, Krzysztof P. Bielawski, Mariusz Grinholc

**Affiliations:** 1 Laboratory of Molecular Diagnostics, Department of Biotechnology, Intercollegiate Faculty of Biotechnology, University of Gdansk and Medical University of Gdansk, Gdansk, Poland; 2 Laboratory of Biophysics, Department of Molecular and Cellular Biology, Intercollegiate Faculty of Biotechnology University of Gdansk and Medical University of Gdansk, Gdansk, Poland

**Keywords:** Antimicrobial resistance, Risk factors, Bacterial infection

## Abstract

Antimicrobial photodynamic inactivation (aPDI) and antimicrobial blue light (aBL) are considered low-risk treatments for the development of bacterial resistance and/or tolerance due to their multitargeted modes of action. In this study, we assessed the development of *Staphylococcus aureus* tolerance to these phototreatments. Reference *S*. *aureus* USA300 JE2 was subjected to 15 cycles of both sub-lethal aPDI (employing an exogenously administered photosensitizer (PS), i.e., rose Bengal (RB)) and sub-lethal aBL (employing endogenously produced photosensitizing compounds, i.e., porphyrins). We demonstrate substantial aPDI/aBL tolerance development and tolerance stability after 5 cycles of subculturing without aPDI/aBL exposure (the development of aPDI/aBL tolerance was also confirmed with the employment of clinical MRSA and MSSA strain as well as other representatives of Gram-positive microbes, i.e. *Enterococcus faecium* and *Streptococcus agalactiae*). In addition, a rifampicin-resistant (RIF^R^) mutant selection assay showed an increased mutation rate in *S*. *aureus* upon sub-lethal phototreatments, indicating that the increased aPDI/aBL tolerance may result from accumulated mutations. Moreover, qRT-PCR analysis following sub-lethal phototreatments demonstrated increased expression of *umuC*, which encodes stress-responsive error-prone DNA polymerase V, an enzyme that increases the rate of mutation. Employment of *recA* and *umuC* transposon *S*. *aureus* mutants confirmed SOS-induction dependence of the tolerance development. Interestingly, aPDI/aBL-tolerant *S*. *aureus* exhibited increased susceptibility to gentamicin (GEN) and doxycycline (DOX), supporting the hypothesis of genetic alterations induced by sub-lethal phototreatments. The obtained results indicate that *S*. *aureus* may develop stable tolerance to studied phototreatments upon sub-lethal aPDI/aBL exposure; thus, the risk of tolerance development should be considered significant when designing aPDI/aBL protocols for infection treatments *in vitro* and in clinical settings.

## Introduction

During the development of new antimicrobial approaches, it is important to assess the risk of tolerance and/or resistance development. Antimicrobial photodynamic inactivation (aPDI) and antimicrobial blue light (aBL) are defined as treatments involving exogenously- or endogenously produced photosensitizing compounds (photosensitizers, PS) that can be activated with light of the appropriate wavelength. This photoactivation leads to the formation of reactive oxygen species (ROS) and free radicals that result in the oxidation of membrane lipids and damage to proteins and nucleic acids^1^. As a consequence, these ROS-triggered damages may lead to microbial cell inactivation and death due to damage to the cell envelops, inactivation of essential proteins and enzymes, and DNA damage.

The following aspects of both phototreatments (aPDI and aBL) allow us to suppose that the development of tolerance is highly probable and that this issue should be thoroughly investigated:

1. DNA damage cannot be considered only a secondary effect of lethal phototreatment as it may occur as one of the primary types of damage induced by sub-lethal aPDI and aBL (evidenced by SOS response activation)^2^.
2. The mutagenicity of oxygen free radicals is unquestionable^3^.
3. aPDI and aBL are treatments that often require repeated applications to successfully eradicate a pathogen at an infection site.
4. Due to the heterogeneous environment of the infection site, it is extremely likely that microorganisms would be exposed to sub-lethal doses of phototreatment; thus, it is questionable whether the damage is sufficient to cause microbial cell death in all phototreated cells and whether the remaining cells may recover and form more tolerant phenotypes.
5. Considering photodynamic therapy (PDT) as an anticancer treatment, several mechanisms of resistance to PDT have already been described^4^.

Tolerance development has been extensively studied separately for both aPDI and aBL and reviewed by Kashef and Hamblin in depth in 2017^5^. All of these studies follow the existing dogma that aPDI and aBL, due to the nonselective, multifactorial and ROS-dependent mechanisms of action of these phototreatments, are unlikely to induce bacterial tolerance and/or resistance. Unfortunately, after critically reviewing the existing published studies, we are convinced that the methodology used for the assessment of the risk of resistance development was not adequate. Indeed, there is no standard protocol to predict bacterial resistance development; however, when introducing new or reviving old antimicrobials, the studies need to meet the following requirements^6–10^:

(i) Test strains need to be exposed to a sub-lethal antimicrobial treatment resulting in a 1 to 3 log_10_ reduction in colony forming units (CFU)/ml to leave sufficient survivors for possible tolerance development^9^.
(ii) The treated bacterial cultures need to be subcultured for up to 15 successive cycles^9^.
(iii) The subculture must originate from the treated suspension (no single surviving colony inoculation is allowed)^8,10^.
(iv) Phenotypic stability testing must be performed^6,9^.

These protocol requirements could have been easily followed when the most recently discovered antimicrobial (i.e., teixobactin) was described^11^. Unfortunately, most phototreatment-related studies do not meet these criteria and instead originate the bacterial culture for the next cycle from a single colony of survivors identified by Petri dish solid medium plating^12–18^. Such an approach is burdened with an extremely low probability of detecting tolerance development as the majority of the surviving population (with possible genetic alterations) is not included in the next cycle. This is likely the key reason why the development of aPDI/aBL tolerance has not been reported.

In addition, when evaluating the risk of resistance development, it is highly important to differentiate the two terms tolerance and resistance. According to the Scientific Committee on Consumer Safety (SCCS)^19^: “the practical meaning of antibiotic resistance is to describe situations where (i) a strain is not killed or inhibited by a concentration attained *in vivo*, (ii) a strain is not killed or inhibited by a concentration to which the majority of strains of that organism are susceptible or (iii) bacterial cells that are not killed or inhibited by a concentration acting upon the majority of cells in that culture” and tolerance denotes a reduced susceptibility to an antimicrobial molecule characterized by an elevated MIC.

The current study aimed to investigate whether the employment of the accepted protocol could detect and predict the risk of aPDI/aBL tolerance development. The obtained results demonstrate that sub-lethal aPDI and aBL treatment leads to tolerance development in *Staphylococcus aureus*. Moreover, an increased mutation rate (rifampicin resistant (RIF^R^) mutant selection), increased stress-responsive error-prone DNA polymerase V gene expression (*umuC*) and lack of tolerance development in *recA*- and *umuC* lacking *S*. *aureus* mutants suggest a possible mechanism of tolerance development. Moreover, data presented justify the assumption of possible aPDI/aBL tolerance development for other Gram-positive and Gram-negative species.

## Materials and Methods

### Experimental workflow

**Figure 1:**
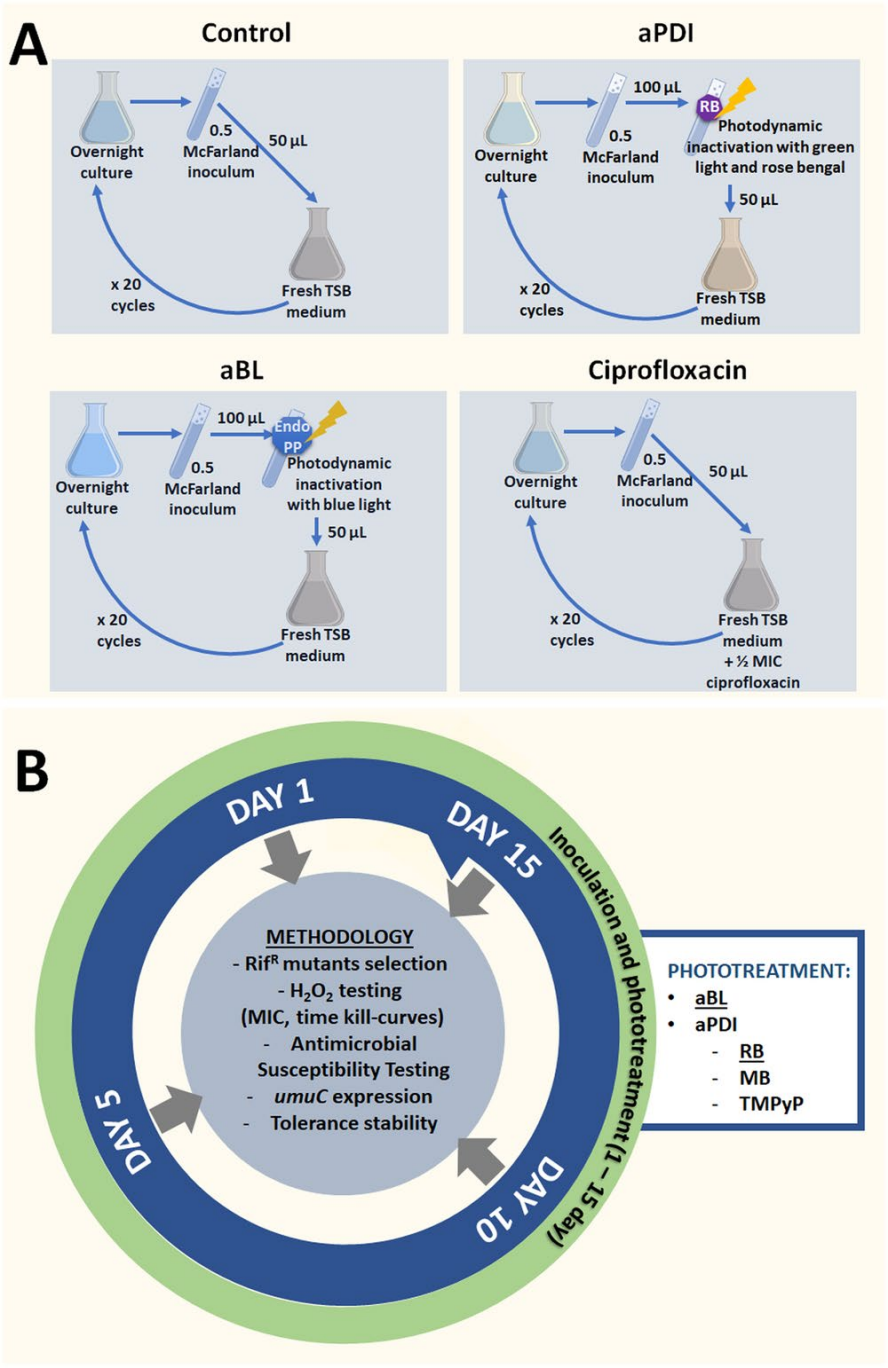
Experimental workflow. The experimental design included the four following treatments (panel A): (i) control samples; (ii) aPDI-treated samples; (iii) aBL-treated samples; and (iv) CIP-treated samples. For the proper implementation of the study, all of the *S*. *aureus* cultures described above (from the 1^st^, 5^th^, 10^th^ and 15^th^ cycles) were tested to demonstrate (panel B): (i) a treatment-induced change in the mutation rate; (ii) a treatment-induced change in the susceptibility to H_2_O_2_; (iii) a treatment-induced change in the susceptibility to selected antimicrobials (GEN, DOX, VAN, CHL and RIF); (iv) whether developed tolerance is limited to the selection treatment or translates to other phototreatments; and (v) the phenotypic stability of the developed tolerance. In addition, whether sub-lethal aPDI and aBL lead to increased *umuC* expression levels was tested.

The experimental design included the four following treatments (Fig. 1A): (i) control samples (i.e., three biologically independent *S*. *aureus* cultures passaged through 15 consecutive cycles with no treatment to assess the naturally occurring rate of mutation and demonstrate whether genetic alterations gained with no selective pressure may induce a detectable level of aPDI/aBL tolerance); (ii) aPDI-treated samples (i.e., three biologically independent *S*. *aureus* cultures treated with a photosensitizer, i.e., RB, and 515 nm light, resulting in sub-lethal inactivation of the microbial population to assess the risk of the development of tolerance to exogenous PS-dependent phototreatment); (iii) aBL-treated samples (i.e., three biologically independent *S*. *aureus* cultures treated with sub-lethal 411 nm blue light to assess the risk of the development of tolerance to phototreatments utilizing endogenously produced photosensitizing compounds, i.e., porphyrins^20^, flavins^21^, adenine dinucleotide (FAD)^22^, nicotinamide adenine dinucleotide (NADH)^22^); and (iv) CIP-treated samples (i.e., three biologically independent *S*. *aureus* cultures treated with a sub-MIC concentration of CIP to serve as a positive control for induced genetic alterations and demonstrate the adequacy and correct execution of employed assays, i.e., RIF^R^ mutant selection and qRT-PCR for the *umuC* expression measurement) according to the studies by Schroder *et al*.^23^. For the proper implementation of the study, all of the *S*. *aureus* cultures described above (from the 1^st^, 5^th^, 10^th^ and 15^th^ cycles) were tested to demonstrate (Fig. 1B): (i) a treatment-induced change in the mutation rate (with the employment of RIF^R^ mutant selection as described by Pope *et al*.^24^); (ii) a treatment-induced change in the susceptibility to H_2_O_2_ (with the measurement of MIC_H2O2_ and time-dependent H_2_O_2_ toxicity, i.e., time-kill curve assay); (iii) a treatment-induced change in the susceptibility to selected antimicrobials (GEN, DOX, VAN, CHL and RIF); (iv) whether developed tolerance is limited to the selection treatment or translates to other phototreatments (the *S*. *aureus* susceptibility to aPDI employing PSs with different structures, i.e., NMB and TMPyP, was profiled); and (v) the phenotypic stability of the developed tolerance (with *S*. *aureus* cultures passaged for 5 cycles without selective pressure). In addition, whether sub-lethal aPDI and aBL lead to increased *umuC* expression levels was tested (to support the probable mechanism of tolerance development).

### Strain and culture conditions

The strains used in the study are listed in Table 1. The strains were cultured at 37 °C in 5 mL tryptic soy broth (TSB, bioMérieux, France) for 16–20 h under aerobic conditions in an orbital incubator (Innova 40, Brunswick, Light source Germany) at 150 rpm.

**Table 1:** Strains used in this study.

Strain	Description	Source, reference
USA300 JE2	CA-MRSA USA300 JE2 strain, derived from USA300 LAC	NARSA
NE805	Mutant derived from USA300 JE2 strain deposited in Nebraska Transposon Mutant Library (NTML) with disrupted *recA* (recombinase A) gene by *bursa aurealis Tn* insertion	NARSA
NE445	Mutant derived from USA300 JE2 strain deposited in Nebraska Transposon Mutant Library (NTML) with disrupted *umuC* gene by *bursa aurealis Tn* insertion	NARSA^59^
1631	MSSA clinical isolate	This study
2030	HA-MRSA clinical isolate	This study
ATCC 27956	*Streptococcus agalactiae* reference strain	^60^
EU87	*Enterococcus faecium* clinical isolate with extensively drug resistance profile (XDR)	This study

### Light source

Irradiation was performed with three light-emitting diode (LED) light sources that emitted blue (λ_max_ 411 nm, an electrical power of 120 W and an irradiance of 130 mW/cm^2^), red (λ_max_ 632 nm, an electrical power of 50 W and an irradiance of 20 mW/cm^2^) and green (λ_max_ 515 nm, an electrical power of 81 W and an irradiance of 70 mW/cm^2^) light (SecureMedia, Poland). The full characteristics of the light sources were published previously^25^.

### PSs

The PSs were purchased from Sigma-Aldrich (Germany). Rose Bengal (RB), 5,10,15,20-tetrakis(1-methyl-4-pyridinio)porphyrin tetra(*p*-toluenesulfonate) (TMPyP) and new methylene blue (NMB) were dissolved in sterile water at a 1 mM concentration and kept in the dark at −20 °C.

### Determination of the minimum inhibitory concentrations (MICs) of antibacterial agents by broth microdilution

According to the European Committee for Antimicrobial Susceptibility Testing (EUCAST), the MIC values of ciprofloxacin (CIP), rifampicin (RIF), chloramphenicol (CHL), vancomycin (VAN), doxycycline (DOX), and gentamycin (GEN) (Sigma-Aldrich, Germany) were tested by the microbroth dilution method^26^.

### Determination of the aPDI/aBL lethal and sub-lethal conditions

Microbial overnight cultures were adjusted to an optical density (OD) of 0.5 McFarland (McF) (5 × 10^7^ CFU/ml) and aliquots of 100 µl per well were transferred to 96-well microtiter plates. A PS was added to the bacterial suspensions at a final concentration of up to 1 µM (RB) or up to 50 µM (TMPyP and NMB). The aPDI samples treated with RB, TMPyP and NMB were incubated at room temperature in the dark for 15 min and then exposed to different light doses up to 40 J/cm^2^ (λ_max_ 515 nm) for RB or up to 65 J/cm^2^ (λ_max_ 632 nm) for TMPyP and NMB. The aBL samples without a PS were irradiated with different light doses of blue light up to 220 J/cm^2^ (λ_max_ 411 nm). After illumination, 10 µl aliquots were serially diluted tenfold in phosphate-buffered saline (PBS) to generate dilutions of 10^−1^ to 10^−4^ and streaked horizontally on TSB plates with agar (bioMérieux, France). The TSB agar plates were incubated at 37 °C for 16–20 h, and then colonies were counted to estimate the survival rate. Control groups included cells that were not treated with PSs or light. Each experiment was performed in triplicate.

For the purpose of this study, lethal doses of aPDI/aBL were defined as the treatment that resulted in an approximately ≥3 log_10_ reduction in CFU, and sub-lethal doses were defined as a 2 log_10_ reduction in CFU^20,27–32^.

### Determination of tolerance development following repeated sub-lethal exposures to aPDI

To investigate whether *S*. *aureus* could develop tolerance to aPDI during subsequent treatments, the JE2 strain was inoculated in three independent replicates and cultured at 37 °C in TSB for 16–20 h. Then, the cultures were diluted to an OD of 0.5 McF (5 × 10^7^ CFU/ml). One hundred microliters of bacterial suspension was incubated with 0.1 μM RB in the dark at room temperature for 15 min and illuminated with 515 nm light at a dose of 10 J/cm^2^. Following exposure, 10 μL aliquots of the treated samples were taken to determine the survival rate. The rest of the sample was centrifuged (3 min, 10,000 rcf) and washed with 90 μL of PBS. Fifty microliters of the samples was transferred into fresh TSB medium (5 mL) to re-grow overnight. The next day, after 16–20 h of incubation, the treatment was repeated under the same conditions. The cycle of exposure - regrowth - exposure was repeated 15 times. Potential reductions in the susceptibility to aPDI were examined after the 5^th^, 10^th^ and 15^th^ consecutive cycles at the higher light doses (up to 40 J/cm^2^). Control groups included cells that were not treated with PSs or light but were treated with a sub-MIC dose of CIP (0.25 μg/ml) (regrowth overnight, 15 passages).

### Determination of tolerance development following repeated sub-lethal exposures to aBL

The JE2 strain was inoculated in triplicate and cultured at 37 °C in TSB for 16–20 h. Then, the cultures were diluted to an OD of 0.5 McF (5 × 10^7^ CFU/ml). One hundred microliters of bacterial suspension was irradiated with 411 nm light at a dose of 150 J/cm^2^. Following exposure, 10 μL aliquots of the treated samples were taken to determine the survival rate, and 50 μL of the samples was transferred into fresh TSB medium (5 mL) to regrow overnight. The next day, after 16–20 h of incubation, the treatment was repeated under the same conditions. The cycle of exposure - regrowth - exposure was repeated 15 times. Potential reductions in the susceptibility to aBL were examined after the 5^th^, 10^th^ and 15^th^ consecutive cycles at the higher light doses of light (up to 220 J/cm^2^).

### Stability of the acquired tolerance to aPDI/aBL

The samples from the 10^th^ consecutive cycle of aPDI (with RB) and aBL treatment were transferred to 5 mL of fresh TSB medium and cultured at 37 °C for 16–20 h. Aliquots of 50 μL of the overnight cultures were transferred to another tube containing TSB medium. The cycle of transfer - regrowth - transfer was repeated 5 times. On day 5, the cultures were diluted to an OD of 0.5 McF (5 × 10^7^ CFU/ml) and 100 μL of the bacterial suspensions was irradiated with 515 nm light at a dose up to 40 J/cm^2^ or 411 nm light at a dose up to 220 J/cm^2^. The resulting suspensions were compared with the initial samples from the 10^th^ consecutive cycle and with untreated controls.

### Determination of the spontaneous mutation frequencies associated with RIF resistance following repeated sub-lethal exposures to aPDI, aBL and CIP

Potential increases in the mutation rate were examined after the 5^th^, 10^th^ and 15^th^ consecutive cycles of exposure to aPDI, aBL and CIP in three independent *S*. *aureus* cultures. One hundred microliter aliquots of the overnight cultures from the 1^st^, 5^th^, 10^th^ and 15^th^ cycles were spread on TSB agar plates containing RIF 0.25 μg/mL (4x MIC) and incubated at 37 °C. After 24 h of incubation, CFU were counted, and mutation rates were calculated according to a previously published protocol^23^.

### Toxicity and time-kill assay of hydrogen peroxide (H_2_O_2_)

The MIC of H_2_O_2_ was determined using the broth microdilution method for the 1^st^, 5^th^, 10^th^ and 15^th^ cycle isolates according to the EUCAST guidelines. Moreover, the time-dependent toxicity of 4% H_2_O_2_ was examined. To assess the survival rates, aliquots were taken at 0, 5, 10, 20, 30 and 60 min after the administration of H_2_O_2_ and spread on TSB agar plates to determine the CFU. Each experiment was performed in triplicate.

### Hydrogen peroxide detection

Working reagent (WR) used in Pierce^TM^ quantitative peroxide assay reagent was prepared in accordance to manufacturer’s recommendation (Thermo Fisher Scientific, USA). Overnight cultures of *S*. *aureus* JE2 strain were adjusted to optical density 0.5 McFarland standard, then the photosensitiser (RB) was added followed with 15 min incubation at room temperature, and afterwards, irradiated with sub-lethal dose of aPDI or aBL. Next, 10 µL of WR were added to 100 µL of irradiated sample and the spectrophotometric measurement was performed at the wavelength 570 nm (Wallac 1420 Victor, Perkin Elmer). Additionally, the same experiment was performed for cells treated with aPDI (without photosensitizer), cells treated only with RB and cells alone. For estimation the H_2_O_2_concentration in tested samples, the standard curve was prepared.

### RNA extraction

Overnight (16–20 h) cultures of the *S*. *aureus* JE2 strain were inoculated in triplicate in fresh TSB medium (OD_600_ = 0.1) and cultured at 37 °C. Culture samples were collected during the logarithmic growth phase (OD_600_ = 0.6). Two milliliters of the bacterial suspensions was incubated with a final concentration of 0.1 μM RB in the dark at room temperature for 15 min and illuminated with 515 nm light at a dose of 10 J/cm^2^. For aBL, 2 mL of the bacterial suspensions was directly illuminated (150 J/cm^2^) after achieving logarithmic growth. Total RNA was isolated from the aPDI/aBL-treated cells and untreated control cells immediately after light exposure. The samples were centrifuged (2 min, 4,500 rpm), and the pellets were incubated with 1 ml of Renozol (TRI RNA Extraction Reagent, GenoPlast Biochemicals). Five hundred microliters of glass beads (0.1–0.11 mm) was added (Sartorius Stedim Biotech GmbH), and the samples were homogenized using the MagNA Lyser Instrument (Roche Life Science) (2 cycles of 20 s, 4,500 rpm). Next, the samples were incubated with 200 μL of chloroform (3 min) and centrifuged (15 min, 12,000 g, 4 °C). The top phase containing RNA was gently collected, and 500 μL of isopropanol was added. After 10 min of incubation, the samples were centrifuged (10 min, 12,000 g, 4 °C). The pellets were resuspended in 1 mL of 75% ethanol and vortexed. Following 5 min of centrifugation (7,500 g, 4 °C), the supernatant was gently collected, and the pellets were dried on a clean bench and suspended in 50 μL of RNA-free water (MP Biomedicals, LCC). RNA was purified with the RNA Clean-up Mini Kit (Syngen) following the provided manuals. The concentration of RNA was measured using a NanoDrop ND-1000 (Thermo Fisher Scientific).

### cDNA synthesis and quantitative real-time PCR conditions

Reverse transcription and quantitative RT-PCR were performed in one step with the use of commercially available QuantiTect® SYBR® Green RT-PCR kit (Qiagen, Hamburg, Germany). The expression levels of the *gmk*, *recA* and *umuC* genes were quantified using HotStarTaq DNA polymerase (Qiagen). Eight microliters of RNA (1 ng/μL) was subjected to amplification in a 20-μL volume containing 5 μM each primer (primer sequences were published previously by Schroder *et al*. Schroder^23^), 10 μL of 2x QuantiTect SYBR Green RT-PCR Master Mix and 0.2 μL of QuantiTect RT Mix (Qiagen). Reverse transcription was performed for 30 min at 50 °C. The PCR initial HotStart step was conducted for 15 min at 95 °C to activate the HotStarTaq polymerase and denature the template DNA. The following cycling conditions were used for the reaction: amplification and quantification program repeated 30 times (94 °C for 15 s, 55 °C for 30 s and a 30 s extension at 72 °C) with a single fluorescence measurement, a melting curve program (65–95 °C with a heating rate of 0.2 °C per second and continuous fluorescence measurement) and finally a cooling step at 4 °C. The specificities of the PCR products were confirmed by an analysis of the dissociation curves. Relative fold change in gene expression was calculated using the 2^−∆∆Ct^ method; *gmk* was used as the internal control.

### Statistical analysis

Statistical analysis was performed in Statistica 12.0 (StatSoft, Tulsa, USA). Data were expressed as mean values. To assess differences between groups, one-way or two-way ANOVA was applied, followed by post-hoc Tukey’s HSD test. To compare categorical variables, Kruskal-Wallis test was used. P values less than 0.05 were considered statistically significant.

### Ethical approval

The manuscript contains no data concerning animal studies, studies involving human subjects or inclusion of identifiable human data or clinical trials; thus, no ethical approval was required.

## Results

### Sub-lethal aPDI/aBL treatment selection

**Figure 2:**
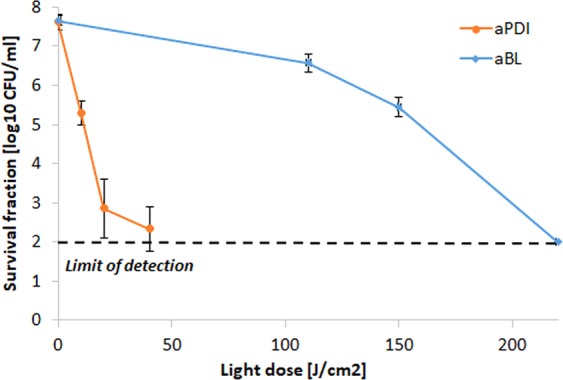
Influence of aPDI and aBL on *S*. *aureus* strain. Microbial overnight cultures were (5 × 10^7^ CFU/ml) were treated with 1 µM RB and exposed to different light doses up to 40 J/cm^2^ (λ_max_ 515 nm). The aBL samples were irradiated with different light doses of blue light up to 220 J/cm^2^ (λ_max_ 411 nm). After illumination, samples were serially diluted, streaked horizontally, incubated at 37 °C for 16–20 h, and then colonies were counted. Control groups included cells that were not treated with PSs or light. For the purpose of this study, lethal doses of aPDI/aBL were defined as the treatment that resulted in an approximately ≥3 log_10_ reduction in CFU, and sub-lethal doses were defined as a 2 log_10_ reduction in CFU. The detection limit was 100 CFU/ml. The values are the means of three separate experiments. Error bars represent standard deviation (SD).

The proper implementation of the accepted protocol for tolerance development risk assessment uses the application of sub-lethal treatment conditions, resulting in reductions in cell viability by 1 to 3 log_10_ units. To meet this requirement, the aPDI and aBL treatment conditions that led to an approximately 2 log_10_ unit reduction in the viable count were defined as sub-lethal and selected for the tolerance development study (Fig. 2). These conditions were 0.1 μM RB photoactivated with green light with a fluence of 10 J/cm^2^, or blue light with a fluence of 150 J/cm^2^ (for aPDI and aBL, respectively).

### RB-aPDI tolerance development was induced only with the RB-aPDI sub-lethal treatment

**Figure 3:**
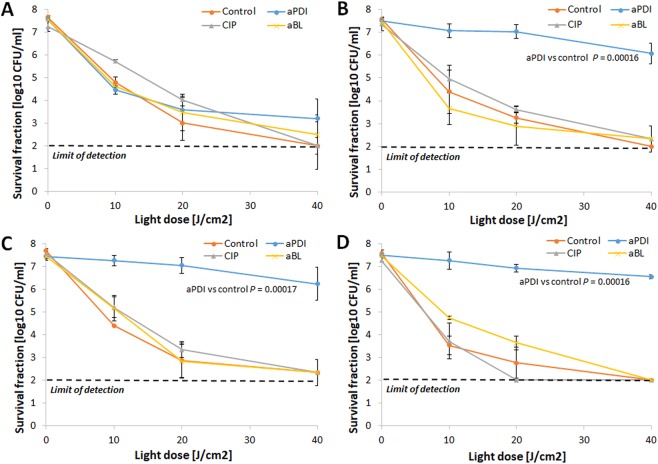
aPDI tolerance development upon sub-lethal RB-aPDI treatment. Overnight JE2 cultures (5 × 10^7^ CFU/ml) were incubated with 0.1 μM RB and illuminated with 515 nm light at a dose of 10J/cm^2^. Following exposure, 10 μL aliquots of the treated samples were taken to determine the survival rate. The rest of the sample was centrifuged and washed with PBS. Fifty microliters of the samples was transferred into fresh TSB medium (5 mL) to re-grow overnight. The next day, the treatment was repeated under the same conditions. The cycle of exposure - regrowth - exposure was repeated 15 times. Control groups included cells that were not treated with PSs or light but were treated with a sub-MIC dose of CIP (0.25 μg/ml) (regrowth overnight, 15 passages). The susceptibility of *S*. *aureus* to RB-aPDI was investigated upon 1 (panel A), 5 (panel B), 10 (panel C) and 15 (panel D) cycles of sub-lethal RB-aPDI/aBL or CIP (½ MIC). The detection limit was 100 CFU/ml. The values are the means of three separate experiments. Error bars represent standard deviation (SD).

When the bactericidal efficacy of RB-dependent aPDI was investigated against both *S*. *aureus* control samples and *S*. *aureus* treated with 15 cycles of RB-aPDI, aBL or CIP, significant tolerance development was observed exclusively in the *S*. *aureus* that underwent the RB-aPDI treatment (Fig. 3). No induction of aPDI tolerance was reported when the selective pressure constituted aBL or CIP treatment nor was aPDI tolerance detected in the control samples. The developed tolerance was detected starting in the 5^th^ consecutive cycle and expressed as a reduction in aPDI antimicrobial efficacy of approximately 3 log_10_ units (Fig. 3B–D). However, the significant reduction in *S*. *aureus* susceptibility to aPDI could be observed starting from 3^rd^ and 4^th^ consecutive cycles (Supplementary Data 1).

To exclude that the green light alone may stand for the observed tolerance development, the appropriate control studies has been included. Five consecutive cycles of green light dose determining sub-lethal aPDI, i.e., 10 J/cm^2^, were applied to *S*. *aureus* JE2. No tolerance development was reported upon light alone treatment indicating the crucial role of ROS generated upon aPDI in tolerance acquisition (Supplementary Data 2).

### aBL tolerance development was induced only with the aBL sub-lethal treatment

**Figure 4:**
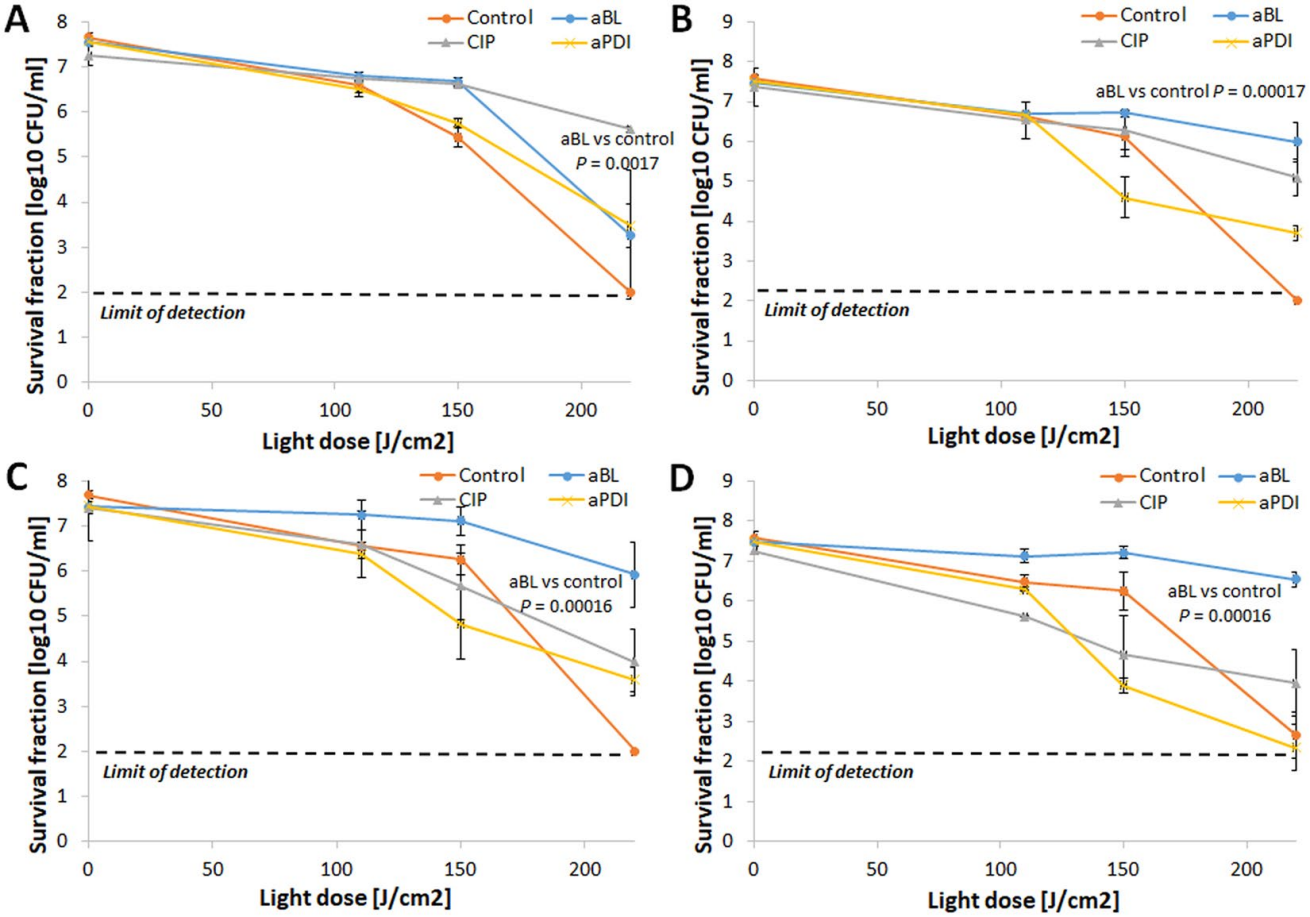
aBL tolerance development upon sub-lethal aBL treatment. Overnight JE2 cultures (5 × 10^7^ CFU/ml) were irradiated with 411 nm light at a dose of 150 J/cm^2^. Following exposure, 10 μL aliquots of the treated samples were taken to determine the survival rate, and 50 μL of the samples was transferred into fresh TSB medium (5 mL) to regrow overnight. The next day, the treatment was repeated under the same conditions. The cycle of exposure - regrowth - exposure was repeated 15 times. The susceptibility of *S*. *aureus* to aBL was investigated upon 1 (panel A), 5 (panel B), 10 (panel C) and 15 (panel D) cycles of sub-lethal aBL/RB-aPDI or CIP (½ MIC). The detection limit was 100 CFU/ml. The values are the means of three separate experiments. Error bars represent standard deviation (SD).

**Figure 5:**
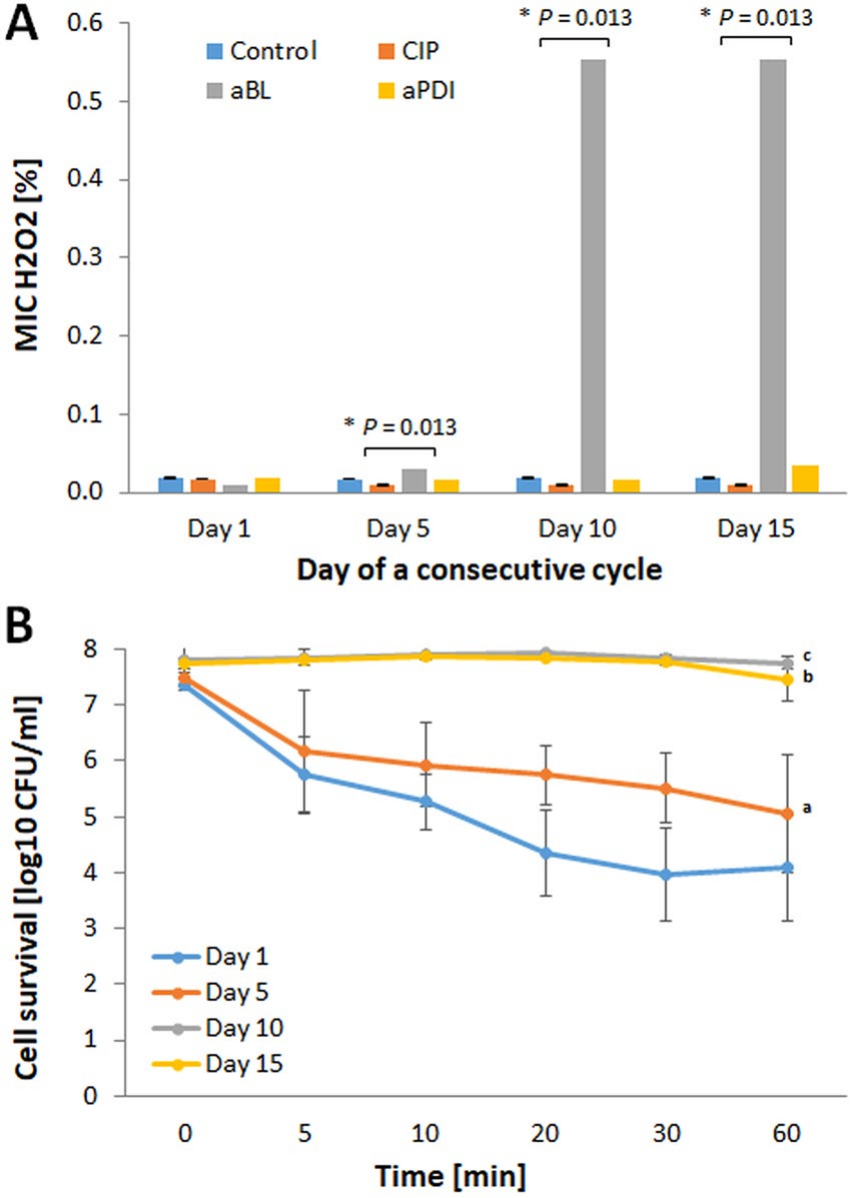
*S*. *aureus* H_2_O_2_ tolerance upon sub-lethal aBL/aPDI and CIP treatment. The MIC of H_2_O_2_ was determined using the broth microdilution method for the 1^st^, 5^th^, 10^th^ and 15^th^ cycle isolates according to the EUCAST guidelines. Moreover, the time-dependent toxicity of 4% H_2_O_2_ was examined. To assess the survival rates, aliquots were taken at 0, 5, 10, 20, 30 and 60 min after the administration of H_2_O_2_ and spread on TSB agar plates to determine the CFU. Hydrogen peroxide MIC was presented at panel A and time-dependent cytotoxicity at panel B. The values are the means of three separate experiments. Error bars represent standard deviation (SD). ^a^ - statistically significant difference from day 1 (*P* = 0.00023), day 10 and day 15 (both *P* = 0.00017), ^b^ - statistically significant difference from day 1 and day 5 (both *P* = 0.00017), ^c^ - statistically significant difference from day 1 and day 5 (both *P* = 0.00017).

The assessment of aBL bactericidal efficacy indicated that *S*. *aureus* aBL tolerance could be developed only upon consecutive sub-lethal aBL treatments (Fig. 4). Neither aPDI nor CIP treatment led to aBL tolerance in *S*. *aureus*. The 2 log_10_ unit decrease in aBL antimicrobial efficacy could be observed starting in the 5^th^ cycle of the consecutive treatments (Fig. 4B,C,D). However, the significant reduction in *S*. *aureus* susceptibility to aBL could be observed starting from 4^th^ consecutive cycle (Supplementary Data 3). Interestingly, the developed aBL tolerance was associated with reduced *S*. *aureus* susceptibility to H_2_O_2_. Both the marked increase in MIC_H2O2_ observed in the 10^th^ and 15^th^ consecutive cycles (Fig. 5A) and the considerable decrease in *S*. *aureus* susceptibility to H_2_O_2_ demonstrated in the time-kill assay (Fig. 5B) indicate that H_2_O_2_ may play a role in the ROS-dependent inactivation induced by aBL treatment.

To support our thesis that H_2_O_2_ plays a significant role in the ROS-dependent inactivation induced by aBL treatment, the amount of H_2_O_2_ produced upon sub-lethal aBL and aPDI treatment has been measured using Pierce^TM^ quantitative peroxide assay. Obtained results indicate that hydrogen peroxide is effectively generated upon aBL treatment, and only barely measurable amounts of H_2_O_2_ are formed upon aPDI exposure (Table 2).

**Table 2:** Hydrogen peroxide measurement.

Conditions	Hydrogen peroxide concentration [µmol/L] (±SD)
Sub-lethal aBL	111.6 (0.71)
Sub-lethal aPDI	1.1 (1.28)
aPDI (without RB)	0 (0.00)
RB (dark)	0.5 (0.48)
Control	14.4 (2.06)

### Developed tolerance was limited to the selection treatment

**Figure 6:**
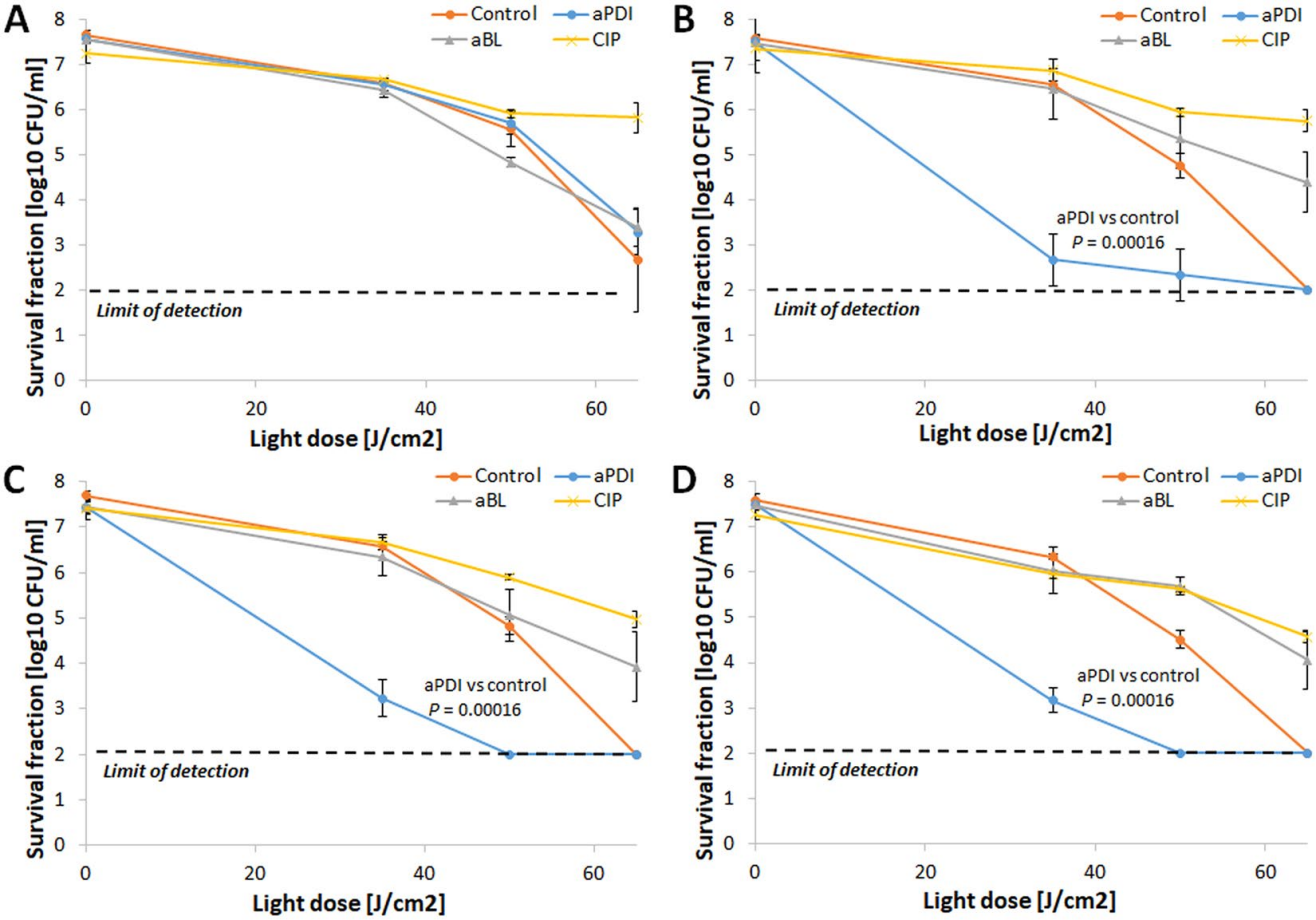
No TMPyP-aPDI tolerance development upon sub-lethal RB-aPDI/aBL or CIP treatment. The JE2 overnight cultures (5 × 10^7^ CFU/ml) originated from 1^st^, 5^th^, 10^th^ and 15^th^ cycles of sub-lethal RB-aPDI/aBL or CIP (½ MIC) treatments were applied to TMPyP-aPDI. The susceptibility of *S*. *aureus* to TMPyP-aPDI was investigated upon 1 (panel A), 5 (panel B), 10 (panel C) and 15 (panel D) cycles of sub-lethal aBL/RB-aPDI or CIP (½ MIC). Microbial overnight cultures were adjusted to an optical density of 5 × 10^7^ CFU/ml and treated with 50 µM TMPyP and exposed to different light doses up to 65 J/cm^2^ (λ_max_ 632 nm). After illumination, 10 µl aliquots were serially diluted to determine survival rate. The detection limit was 100 CFU/ml. The values are the means of three separate experiments. Error bars represent standard deviation (SD).

**Figure 7:**
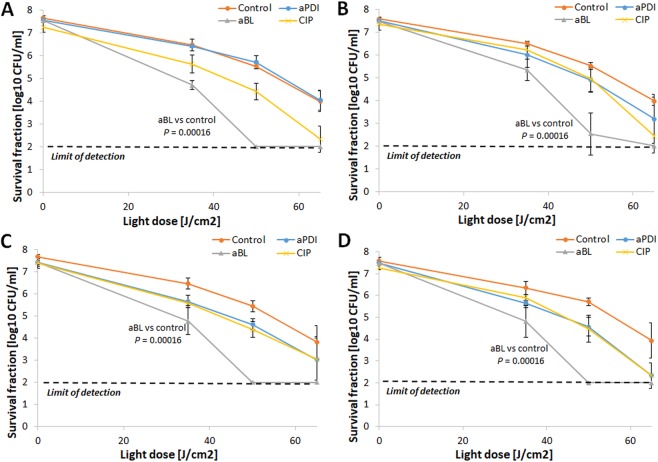
No NMB-aPDI tolerance development upon sub-lethal RB-aPDI/aBL or CIP treatment. The JE2 overnight cultures (5 × 10^7^ CFU/ml) originated from 1^st^, 5^th^, 10^th^ and 15^th^ cycles of sub-lethal RB-aPDI/aBL or CIP (½ MIC) treatments were applied to NMB-aPDI. The susceptibility of *S*. *aureus* to NMB-aPDI was investigated upon 1 (panel A), 5 (panel B), 10 (panel C) and 15 (panel D) cycles of sub-lethal aBL/RB-aPDI or CIP (½ MIC). Microbial overnight cultures were adjusted to an optical density of 5 × 10^7^ CFU/ml and treated with 50 µM NMB and exposed to different light doses up to 65 J/cm^2^ (λ_max_ 632 nm). After illumination, 10 µl aliquots were serially diluted to determine survival rate. The detection limit was 100 CFU/ml. The values are the means of three separate experiments. Error bars represent standard deviation (SD).

To investigate whether the developed tolerance could translate into reduced *S*. *aureus* susceptibility to aPDI employing PSs with different structures, the *S*. *aureus* responses to TMPyP- and NMB-aPDI were investigated for all selection treatments (Figs 6 and 7). The obtained results indicate that 15 consecutive cycles of both sub-lethal RB-aPDI and aBL could not induce *S*. *aureus* tolerance to phototreatments employing other photosensitizing compounds, i.e., NMB and TMPyP. Interestingly, RB-aPDI-treated *S*. *aureus* exhibited even higher susceptibility to TMPyP-mediated aPDI, resulting in an approximately 4 log_10_ increase in TMPyP-aPDI antimicrobial efficacy starting in the 5^th^ consecutive cycle (Fig. 6A–D). No reduction in efficacy of NMB-mediated aPDI was reported after 15 cycles of RB-aPDI, aBL and CIP treatment, though, it seems that particularly aBL treated *S*. *aureus* expressed even higher susceptibility to NMB-aPDI (Fig. 7A–D).

### Developed aPDI/aBL-tolerant phenotype is stable

**Figure 8:**
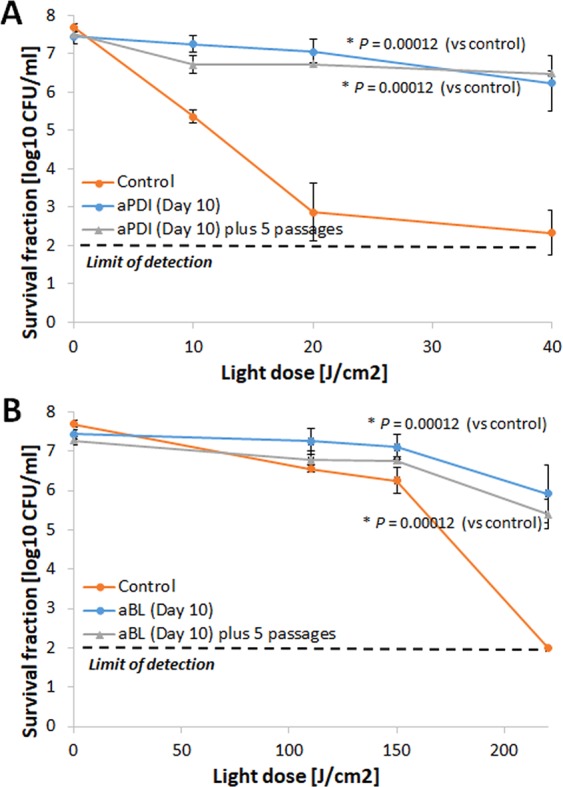
Phototolerant phenotype stability. The samples from the 10^th^ consecutive cycle of aPDI (with RB) and aBL treatment were transferred to 5 mL of fresh TSB medium and cultured at 37 °C for 16–20 h. Aliquots of 50 μL of the overnight cultures were transferred to another tube containing TSB medium. The cycle of transfer - regrowth - transfer was repeated 5 times. On day 5, the cultures (5 × 10^7^ CFU/ml) were irradiated with 515 nm light at a dose up to 40 J/cm^2^ or 411 nm light at a dose up to 220 J/cm^2^. The resulting suspensions were compared with the initial samples from the 10^th^ consecutive cycle and with untreated controls.The susceptibility of *S*. *aureus* to phototreatment was investigated for *S*. *aureus* treated with 10 consecutive cycles of sub-lethal aPDI (panel A) and aBL (panel B) and compared when the tolerant strain was administered 5 additional passages with no selection treatment. The detection limit was 100 CFU/ml. The values are the means of three separate experiments. Error bars represent standard deviation (SD).

The phenotypic stability of the developed tolerance is one of the crucial issues when assessing the risk of resistance development; thus, *S*. *aureus* cultures expressing significant tolerance to the studied phototreatments (i.e., RB-aPDI and aBL) that originated from the 10^th^ consecutive cycle were passaged for the next five cycles with no selection pressure. Afterwards, the susceptibility of the passaged *S*. *aureus* cultures to RB-aPDI and aBL was investigated and compared with the susceptibility of *S*. *aureus* originating directly from the 10^th^ cycle of treatment. No loss of the developed aPDI/aBL tolerance was reported (Fig. 8A,B). The obtained results support the assumption that developed tolerance results from genetic alterations induced by multiple sub-lethal treatments.

### Genetic alterations in *S*. *aureus* may be induced by sub-lethal aPDI/aBL

**Figure 9:**
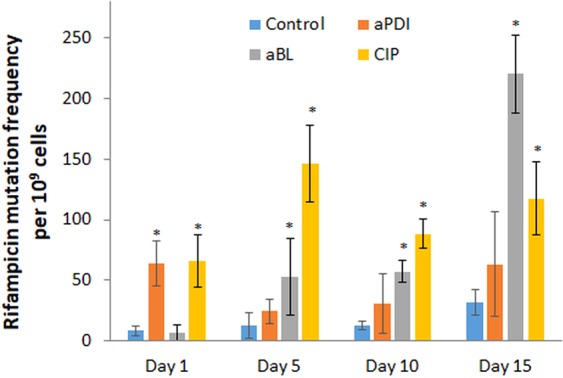
Effect of sub-lethal phototreatments and CIP on the mutation rate. Potential increases in the mutation rate were examined after the 5^th^, 10^th^ and 15^th^ consecutive cycles of exposure to aPDI, aBL and CIP (½ MIC). One hundred microliter aliquots of the overnight cultures from the 1^st^, 5^th^, 10^th^ and 15^th^ cycles were spread on TSB agar plates containing RIF 0.25 μg/mL (4x MIC) and incubated at 37 °C. After 24 h of incubation, CFU were counted, and the mutation frequency was defined as the ratio of RIF^R^ colonies in relation to the total number of bacteria (CFU). The bars represent the mean values of three biological replicates ± standard deviation (SD). *P < *0*.*05* (*vs control*).

To support the finding concerning the phenotypic stability of the developed tolerance, we aimed to investigate whether sub-lethal aPDI/aBL treatment may lead to an increased mutation rate in *S*. *aureus*. As *S*. *aureus* resistance to RIF may result from a single spontaneous mutation in *rpoB*, the RIF^R^ mutant selection assay is considered an adequate methodology for mutation rate estimation. The obtained results indicate that, both sub-lethal aPDI and sub-lethal aBL as well as CIP (positive control) lead to a significantly increased mutation rate in *S*. *aureus* (Fig. 9).

**Figure 10:**
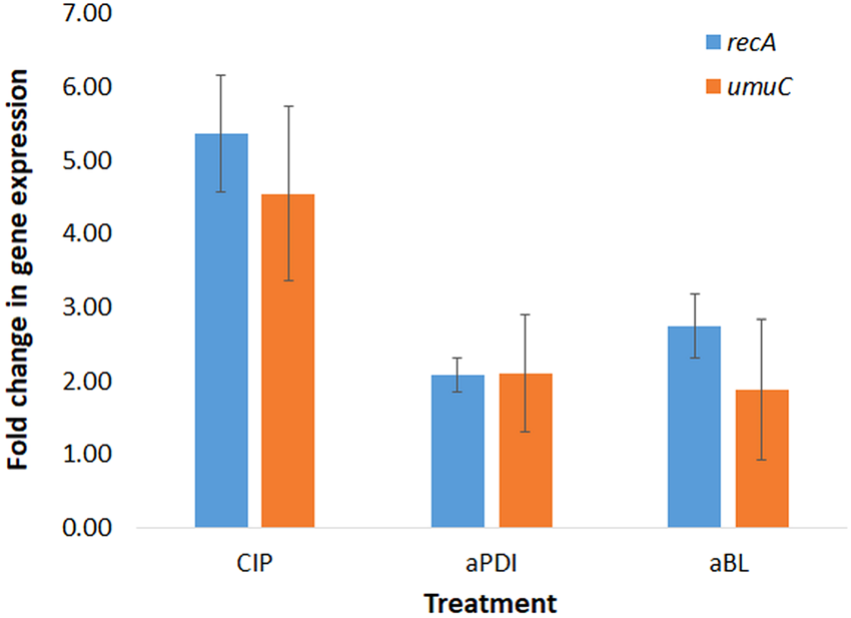
Effect of aPDI, aBL and ciprofloxacin on *recA* and *umuC* expression. *S*. *aureus* was grown to exponential phase (OD_600_ 0.6) and treated with sub-lethal aPDI/aBL or CIP (½ MIC) for 1 h. Relative expression of *recA* and *umuC* in relation to *gmk* was assessed by qRT-PCR. Bars represent mean values of at least three biological replicates + SD.

We assumed that this increased mutation rate could result directly from induced DNA damage, as evidenced in our previously published report^2^, but may also result from the activity of stress-responsive error-prone DNA polymerase V, as evidenced by the increased expression of *umuC* in response to both sub-lethal phototreatments (Fig. 10). To confirm that employed treatments conditions do activate SOS response, the expression level of *recA* was also investigated and demonstrated to be increased by sub-lethal phototreatments (Fig. 10).

**Figure 11:**
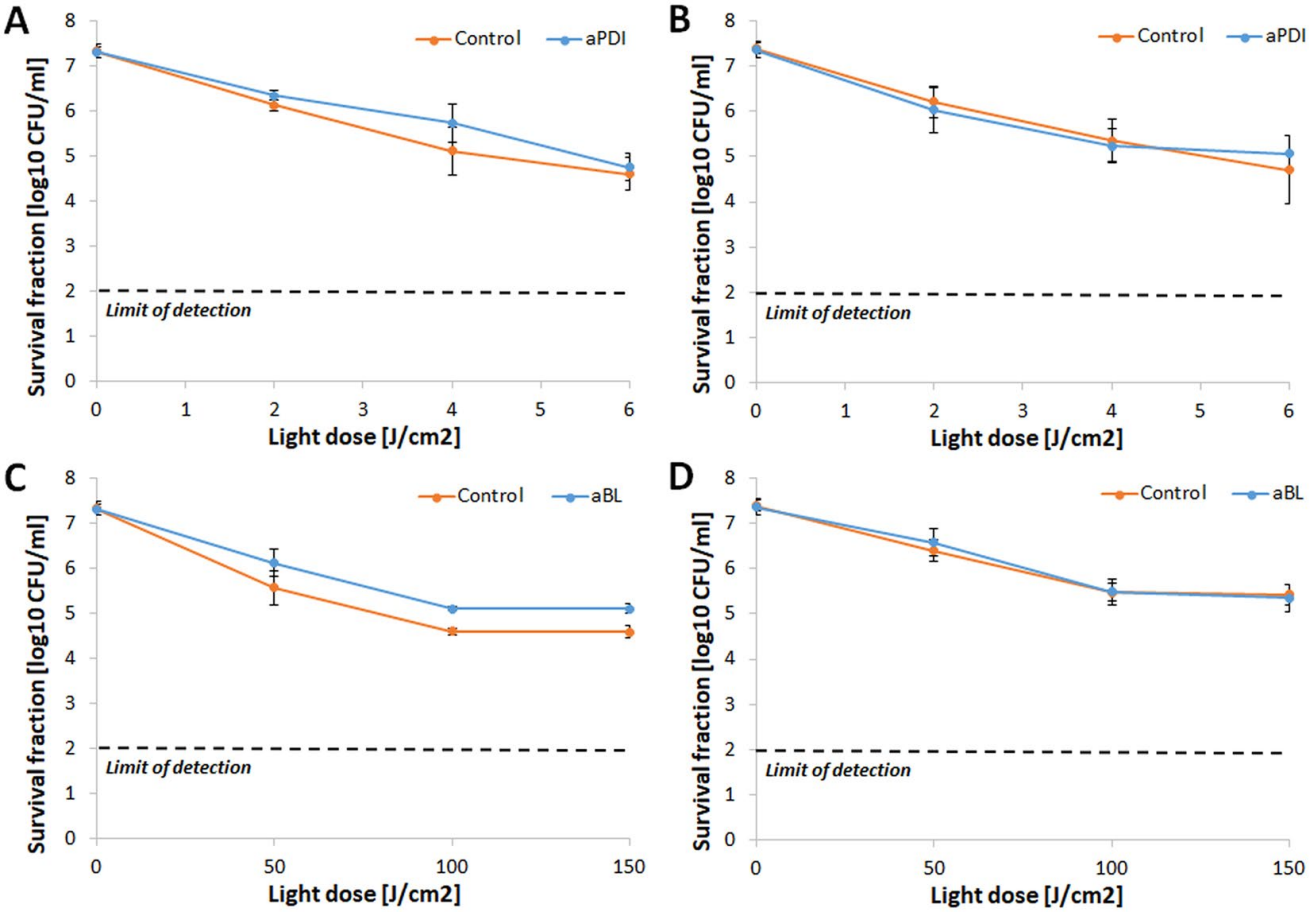
Lack of tolerance development upon sub-lethal aPDI/aBL treatment in *recA*-deficient *S*. *aureus* mutant. Overnight JE2 SE805 cultures (5 × 10^7^ CFU/ml) were incubated with 0.1 μM RB and illuminated with 515 nm light at a dose of 2 J/cm^2^. Following exposure, 10 μL aliquots of the treated samples were taken to determine the survival rate. The rest of the sample was centrifuged and washed with PBS. Fifty microliters of the samples was transferred into fresh TSB medium (5 mL) to re-grow overnight. The next day, the treatment was repeated under the same conditions. The cycle of exposure - regrowth - exposure was repeated 5 times. In case of aBL treatment, overnight cultures were irradiated with 411 nm light dose of 50 J/cm^2^. The susceptibility of *S*. *aureus* to RB-aPDI was investigated upon 1 (panel A) and 5 (panel B) cycles of sub-lethal RB-aPDI, and susceptibility of *S*. *aureus* to aBL was investigated upon 1 (panel C) and 5 (panel D) cycles of sub-lethal aBL. The detection limit was 100 CFU/ml. The values are the means of three separate experiments. Error bars represent standard deviation (SD).

To support our thesis of possible SOS-dependent mechanism underlying developed tolerance, we have employed *recA* and *umuC* transposon *S*. *aureus* mutants within the current study. Obtained results do indicate that aPDI as well as aBL tolerance development may be considered *recA*-dependent processes as *S*. *aureus* lacking functional *recA* gene expressed no tolerance to both phototreatments upon five consecutive sub-lethal aPDI (Fig. 11A,B) and aBL (Fig. 11C,D) cycles.

**Figure 12:**
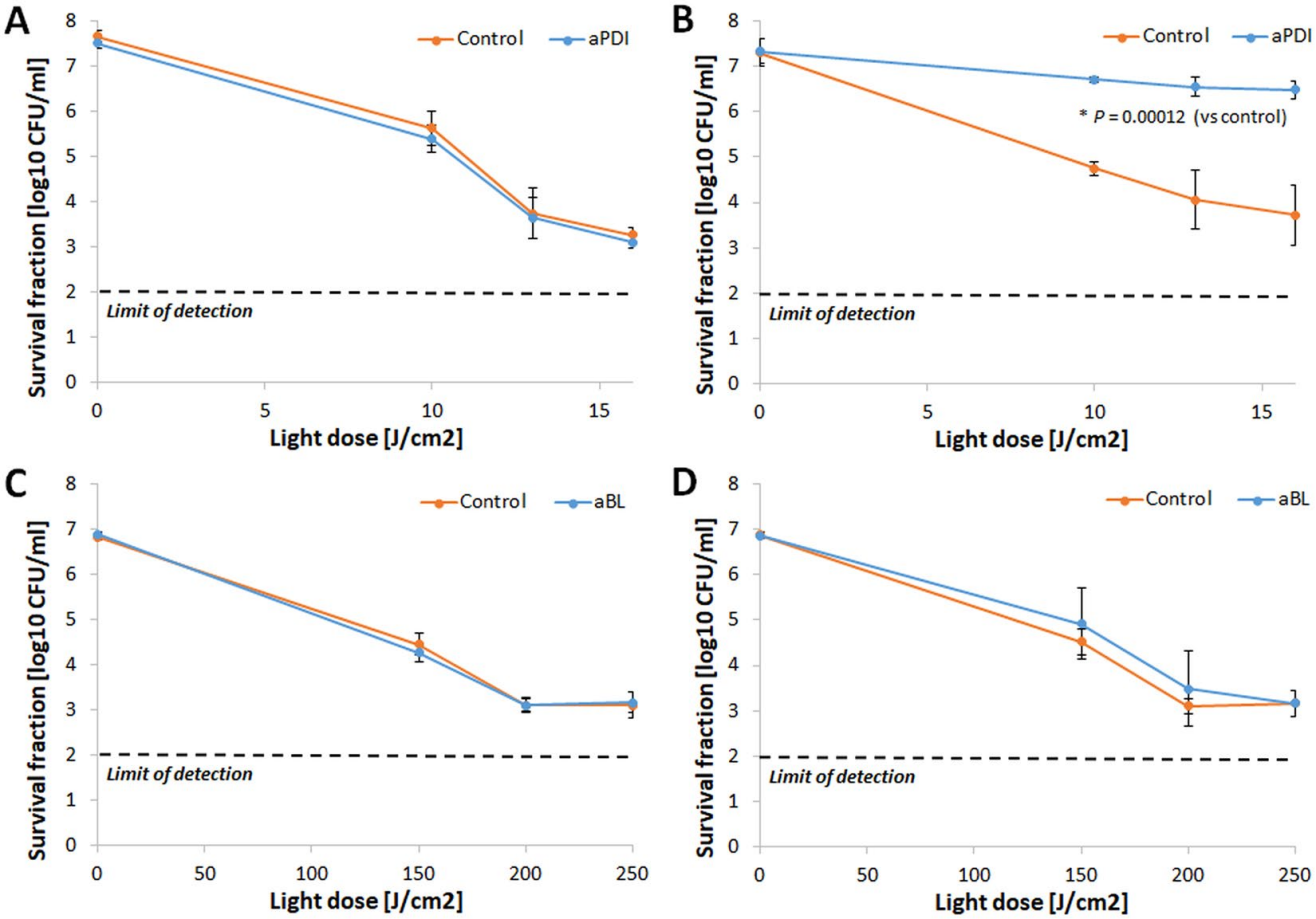
aBL tolerance development is *umuC*-dependent. Overnight JE2 SE445 cultures (5 × 10^7^ CFU/ml) were incubated with 0.1 μM RB and illuminated with 515 nm light at a dose of 10 J/cm^2^. Following exposure, 10 μL aliquots of the treated samples were taken to determine the survival rate. The rest of the sample was centrifuged and washed with PBS. Fifty microliters of the samples was transferred into fresh TSB medium (5 mL) to re-grow overnight. The next day, the treatment was repeated under the same conditions. The cycle of exposure - regrowth - exposure was repeated 5 times. In case of aBL treatment, overnight cultures were irradiated with 411 nm light dose of 150 J/cm^2^. The susceptibility of *S*. *aureus* to RB-aPDI was investigated upon 1 (panel A) and 5 (panel B) cycles of sub-lethal RB-aPDI, and susceptibility of *S*. *aureus* to aBL was investigated upon 1 (panel C) and 5 (panel D) cycles of sub-lethal aBL. The detection limit was 100 CFU/ml. The values are the means of three separate experiments. Error bars represent standard deviation (SD).

Interestingly, the tolerance development studies applied to *umuC* transposon *S*. *aureus* mutant revealed that only aBL tolerance acquisition is considered *umuC*-dependent process. *S*. *aureus* NE445 lacking functional *umuC* gene developed significant aPDI tolerance (Fig. 12A,B) and expressed no aBL tolerance upon five cycles of sub-lethal aPDI and aBL, respectively (Fig. 12C,D).

### Mult**i**ple sub-lethal aPDI/aBL and CIP treatments lead to *S*. *aureus* sensitization to GEN and DOX

**Figure 13:**
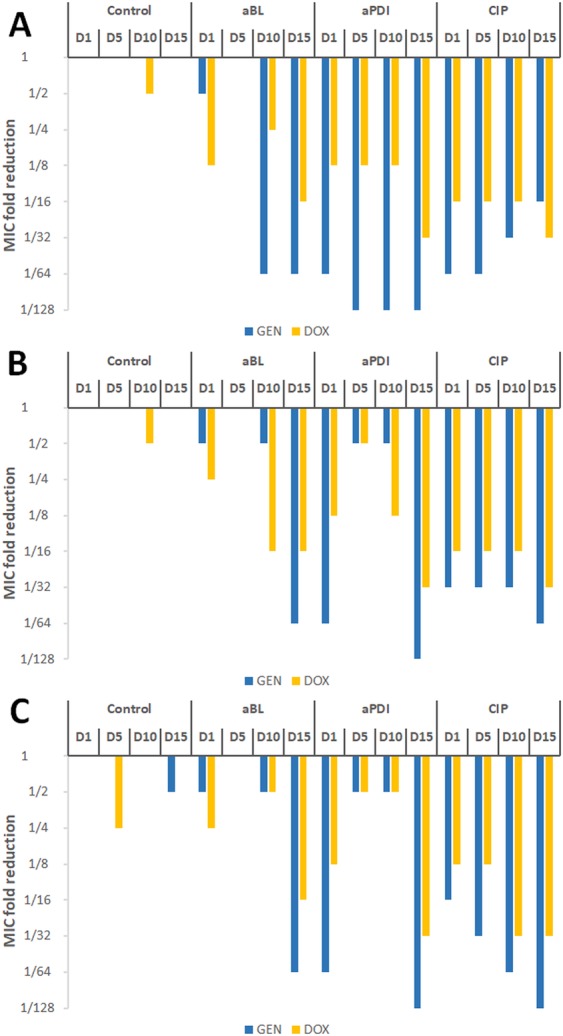
Influence of sub-lethal aPDI/aBL and ciprofloxacin on *S*. *aureus* susceptibility to GEN and DOX. *S*. *aureus* was applied with sub-lethal phototreatment and sub-MIC CIP for 15 consecutive cycles followed by antimicrobial susceptibility testing for gentamycin (GEN) and doxycycline (DOX). Panels A-C represents data obtained for three biological independent experiments.

As induced genetic alterations may affect antimicrobial susceptibility, the *S*. *aureus* bacteria subjected to multiple sub-lethal phototreatments and sub-MIC CIP treatments were examined for their susceptibility to selected antimicrobials, i.e., GEN, DOX, VAN, CHL and RIF. The obtained results demonstrate that multiple sub-lethal phototreatments may lead to *S*. *aureus* sensitization to selected antimicrobials (as evidenced for GEN and DOX) (Fig. 13). The same phenomenon was observed in all three biologically independent repetitions (Fig. 13A–C). Upon 15 consecutive cycles of sub-lethal phototreatment, the 32- and 128-fold MIC reductions were observed for DOX and GEN, respectively.

### Developed aPDI/aBL tolerance cannot be considered resistance

**Figure 14:**
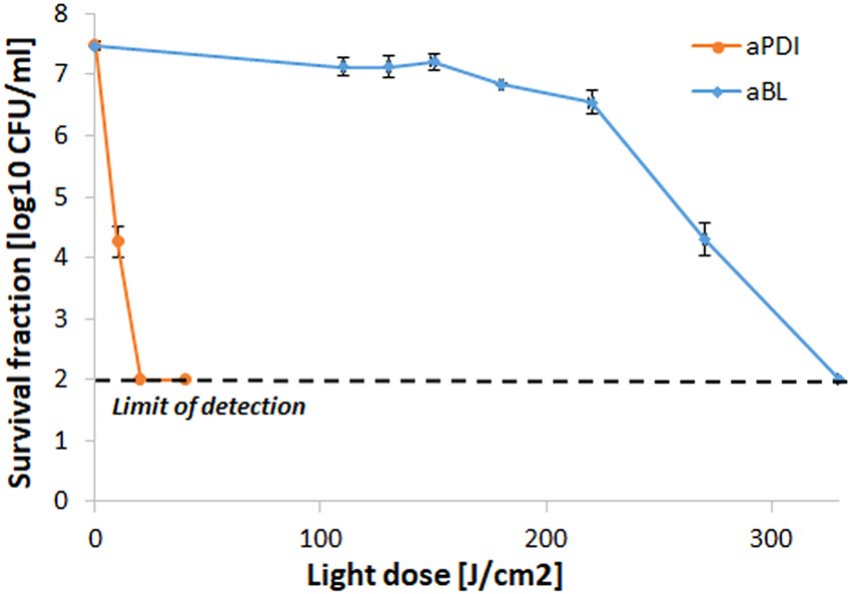
More rigorous aPDI and aBL treatment conditions exert bactericidal effect on *S*. *aureus* phototolerant strain. aPDI treatment of *S*. *aureus*. Light doses ranging from 20 to 130 J/cm^2^ (λ 515 nm) and 1 μM rose Bengal. aBL treatment of *S*. *aureus*. Light doses ranging from 0 to 330 J/cm^2^ (λ 411 nm) were applied. The detection limit was 100 CFU/ml. The values are the means of three separate experiments. Error bars represent standard deviation (SD).

It is significant to underline that the observed decreases in *S*. *aureus* susceptibility to both aPDI and aBL phototreatments cannot be considered resistance, as the employment of more rigorous experimental conditions, i.e., increased PS concentrations and/or higher light doses, results in bacterial eradication (Fig. 14). To demonstrate it, the susceptibility of the aPDI/aBL-tolerant *S*. *aureus* (from the 15^th^ cycle of the treatment) to aPDI and aBL was investigated with the employment of increased light doses (up to 150 J/cm^2^ for the aPDI and up to 330 J/cm^2^ for the aBL) and increased RB concentration of 1 μM.

### Developed tolerance is not limited to CA-MRSA isolates

To support or findings concerning possible aPDI/aBL tolerance development and indicate that the observed tolerance is not restricted only to CA-MRSA isolate, we have employed within the study both MRSA and MSSA clinical isolates as well as two representatives of other Gram-positive microbes, i.e., *Enterococcus faecium* and *Streptococcus agalactiae*. Application of five consecutive cycles od sub-lethal aPDI and aBL resulted in significant tolerance development for all studies strains/species (Table 3). Deeper insight into developed tolerance has been presented within the Supplementary Data indicating bacterial response to aPDI and/or aBL with the administration of various treatment conditions upon 1^st^ and 5^th^ consecutive cycle of sub-lethal treatment (Supplementary Data 4).

**Table 3:** Developed aPDI/aBL tolerance.

Species	Sub-lethal conditions		Developed tolerance^a^	
	aPDI	aBL	aPDI	aBL
*S*. *aureus* (MSSA)	0.1 μM RB green light fluence 8 J/cm^2^	Blue light fluence 50 J/cm^2^	4 log_10_	3 log_10_
*S*. *aureus* (HA-MRSA)	0.1 μM RB green light fluence 10 J/cm^2^	Blue light fluence 50 J/cm^2^	3.5 log_10_	3 log_10_
*S*. *agalactiae*	0.05 μM RB green light fluence 20 J/cm^2^	ND	3 log_10_	—
*E*. *faecium*	0.1 μM RB green light fluence 10 J/cm^2^	Blue light fluence 150 J/cm^2^	2 log_10_	3 log_10_

^a^Expressed as the highest observed reduction in aPDI and aBL efficacy upon five consecutive cycles of sub-lethal treatment. ND, not determined.

## Discussion

**Figure 15:**
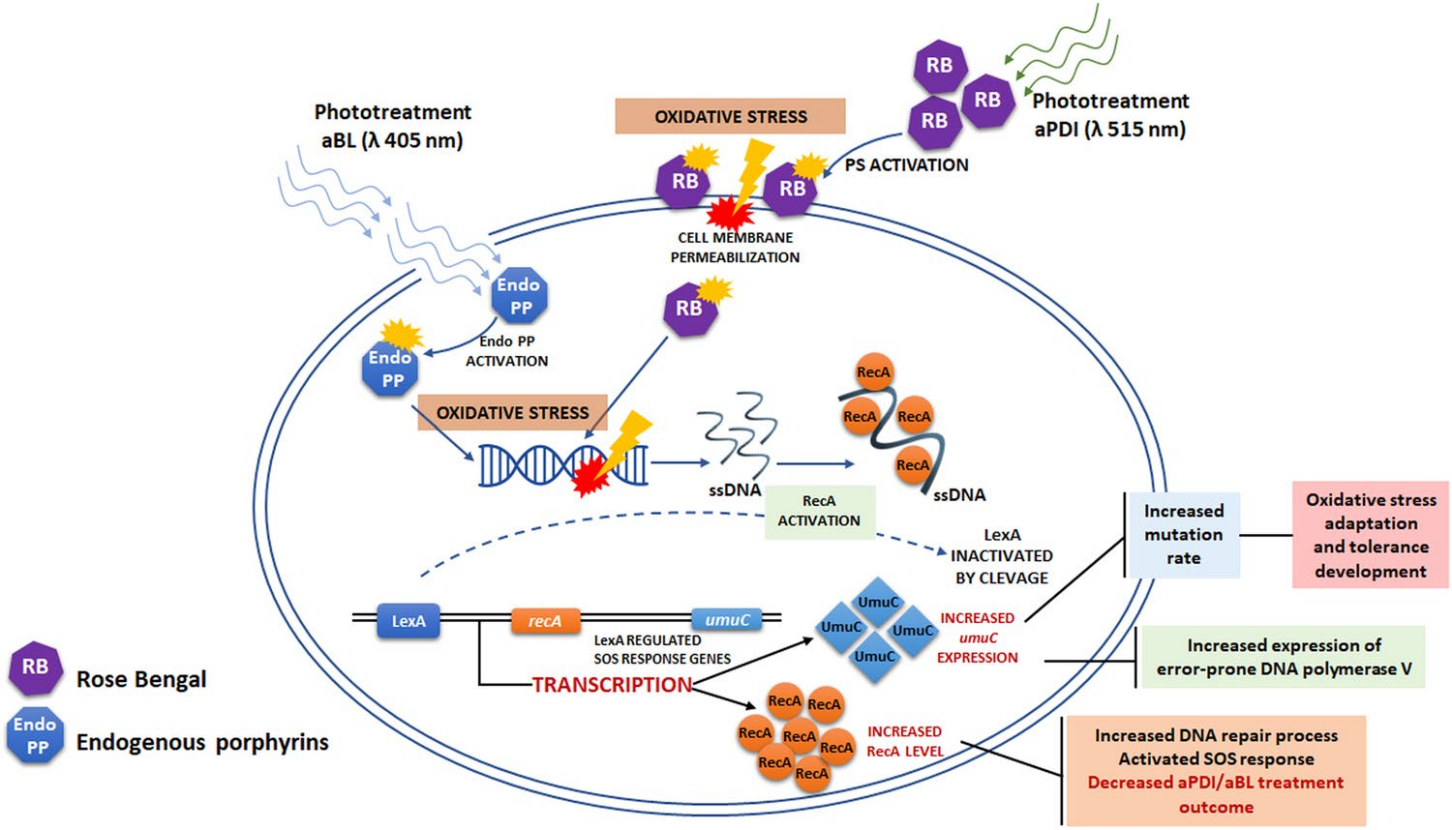
Proposed mechanism underlying the adaptation process. Irradiated sensitizer molecules achieve an activated state and lead to the production of reactive oxygen species as well as free radicals (oxidative stress). This process results in cell-membrane as well as DNA damage. RecA responds to DNA damage by binding to ssDNA, which triggers autocleavage of LexA. The LexA repressor dissociates from the SOS boxes and induces transcription of the SOS regulon, including *recA* and *umuC* expression.

In a previous report, we demonstrated that sub-lethal aBL and aPDI (employing various photosensitizing compounds, i.e., RB, NMB, toluidine blue O (TBO), a cationic porphyrin derivative (TMPyP) and zinc phthalocyanine (ZnPc)) drive substantial DNA damage in *S*. *aureus*, leading to the activation of RecA and an increased SOS response^2^. That study was the foundation for the hypothesis that phototreatments may lead to tolerance development. The aim of this current work was to demonstrate whether tolerance development occurs. The application of the accepted protocol for the evaluation of resistance development as well as the investigation of *umuC* and *recA* expression upon sub-lethal aPDI/aBL treatment demonstrated that *S*. *aureus* may develop tolerance to the phototreatments and indicated the possible mechanism underlying the acquired tolerance (Fig. 15). We are convinced that sub-lethal aPDI/aBL lead to DNA damage in live *S*. *aureus* cells, resulting in the activation of RecA and the SOS response, which translates into increased expression of error-prone DNA polymerase V, the enzyme responsible for the increased mutation rate, and this process drives *S*. *aureus* treatment adaptation. Obviously, aPDI/aBL-induced DNA damage may also be directly responsible for the genetic alterations that lead to the reduced susceptibility of *S*. *aureus* to the treatments.

Our studies indicate that both phototreatments tolerance development are *recA*-dependent processes confirming that photoinduced DNA damage occur upon aPDI/aBL treatments. It is in accordance with previously published studies indicating that ROS generated upon aPDI and aBL treatment, i.e., hydrogen peroxide, as well as singlet oxygen may induce oxidative DNA damage and activate SOS response^33,34^. Though both phototreatments seem to be *recA*-dependent, only aBL tolerance development demonstrated to be *umuC*-dependent process. Possible explanation lays within the significant difference in proportion of H_2_O_2_ and singlet oxygen formed upon aPDI and aBL treatments. The current study demonstrated that the level of H_2_O_2_ produced upon photoinactivation is remarkably higher in case of aBL than aPDI treatment. This finding also provides the explanation for the simultaneous development of aBL and H_2_O_2_ tolerance upon numerous cycles of sub-lethal aBL. Increased MIC of H_2_O_2_ was also reported by Tomb *et al*.^15^ in case of MSSA cultured in the presence of 405 nm light when compared to dark control, providing supportive evidence for our observations. On other hand, the acquired H_2_O_2_ tolerance observed exclusively in case of aBL treated *S*. *aureus* indicates that the various level of hydrogen peroxide production must be considered a significant issue. Contradictory, RB mediated aPDI, with low H_2_O_2_ formation, is considered model photoprocess of singlet oxygen production^35^. This discrepancy in H_2_O_2_ and singlet oxygen production between aPDI and aBL treatments may explain *umuC* dependence of aBL tolerance development. The study by Goerlich *et al*.^33^ demonstrated that H_2_O_2_ appears to induce DNA strand breaks which would be at the origin of SOS induction. Imlay and Linn^36^ supported this finding revealing that mutant strains which cannot induce SOS regulon were hypersensitive to killing by H_2_O_2_. Our findings stating the *recA*-dependence of aBL tolerance are in agreement with described studies and indicate that H_2_O_2_ produced upon aBL treatment may induce DNA breaks and leads to SOS system induction. Moreover, study by Imlay and Linn revealed that H_2_O_2_ induced mutagenesis, and Storz *et al*.^37^ further demonstrated that *umuC* is involved within this process. These observations suggest that H_2_O_2_ induced DNA lesions can be processed by the error-prone DNA repair system which depends on the induction of the SOS response. It supports our findings stating *umuC*-dependence of aBL tolerance development. Opposite effect observed for aPDI tolerance (*umuC*-independence) may be explained with different DNA lesions induced with RB-generated singlet oxygen. Results by Tudek *et al*.^38^ revealed that aPDI with methylene blue (MB), another effective singlet oxygen producer, results mostly in single base substitutions (the most frequent base change is the GC → TA transversion) and induce mutagenesis that is at least partially independent on the induction of SOS response and *umuC*. Epe *et al*.^39^ also evidenced that singlet oxygen is the ultimate reactive species leading to increased mutagenesis by MB and light. The same was reported for RB photosensitization^40^. In addition, singlet oxygen induced mutagenesis was also considered SOS-independent in study by Decuyper-Debergh *et al*.^41^. Our findings are in accordance to these observations and indicate that RB mediated aPDI may result in DNA lesions that lead to increased SOS- and *umuC*-independent mutagenesis. The results described within the current study are not surprising as long as McKenzie *et al*.^42^ confirmed that other than *umuC*, but RecA-dependent SOS-regulated genes are required for adaptive mutation.

A key issue of obtained results is the lack of cross resistance between RB-aPDI and aPDI mediated by other PSs, i.e., NMB and TMPyP, along with lack of cross resistance between aPDI and aBL. As far as our results demonstrated significant discrepancy in the distribution of H_2_O_2_ and singlet oxygen between aBL and aPDI treatments, we assume that this different production of mentioned reactive species may provide the possible explanation for the lack of cross resistance between aPDI and aBL. In case of lack of cross resistance between aPDI involving different PSs we are not able to identify the possible explanation of observed phenomenon. All of employed PSs, i.e., RB, NMB and TMPyP, are considered efficient singlet oxygen producers. The most obvious difference between RB and NMB/TMPyP mediated aPDI is the application of light of different wavelength, 515 instead of 632 nm, respectively. However, obtained results exclude the possibility that the light alone could stand for developed aPDI tolerance. The administration of five consecutive cycles of sub-lethal green light did not affect *S*. *aureus* susceptibility to RB-aPDI indicating that ROS generated upon aPDI treatment are crucial for the tolerance development. We are convinced that multiple variables may be involved in the observed lack of cross resistance between aPDI employing different PSs, i.e., different cellular localization of studied PSs, various specificity for microbial targets (cell envelopes, proteins, genetic material), exerting its deleterious effects against DNA directly through the action of generated ROS and singlet oxygen or indirectly through oxidized membrane lipids etc.); thus, within the current study it is hard to speculate what could be the key component.

Possible aPDI/aBL tolerance development has been extensively studied, and numerous studies have been published previously; however, we find the employed methodologies inappropriate. Most of the studies duplicate the basic mistake of performing a single colony inoculation of the culture to begin the next treatment cycle^12–16^. This approach results in the omission of the majority of the bacterial population and a substantial reduction in the probability of tolerance detection. Two other studies by Amin *et al*.^32^ and Al-Mutairi *et al*.^43^ seem to meet the requirement of using broth inoculation; however, we consider the conclusions of these studies concerning the lack of aPDI/aBL tolerance development exaggerated. According to the study by Amin *et al*., no tolerance development was observed; however, after 9 cycles of sub-lethal aPDI treatment, the bacteria exhibited reduced susceptibility to the treatment as the surviving fraction of the treated suspension was increased by approximately 2 log_10_ units. The authors’ statement probably results from the fact that the observed difference was not statistically significant, nevertheless, in our opinion, this observation indicates possible tolerance development. The study by Al-Mutairi *et al*. stated that sub-lethal aPDI treatment does not lead to the development of resistance; however, according to the presented data, we cannot consider the applied aPDI treatment sub-lethal. As far as we understand, the employed aPDI conditions resulted in an approximately 5 log_10_ unit reduction in the viable count and not the 1–3 log_10_ unit reduction stated.

Our findings demonstrate that *S*. *aureus*, as well as other Gram-positive microbes, may develop tolerance to studied phototreatments. As the developed tolerance could be easily induced for *S*. *agalactiae* and *E*. *faecium*, it justifies the assumption that the same phenomenon should be expected for Gram-negatives that were not included within the current study. There are several possible mechanisms that could be responsible for the decreased susceptibility of *S*. *aureus* to aPDI/aBL. Pomposiello and Demple^44^ revealed that ROS may increase the expression of ROS detoxification enzymes as well as systems leading to oxidative damage repair. In addition, Orlandi *et al*. and Sistrom *et al*. demonstrated the role of pigments in increased tolerance to aPDI-induced oxidative stress^45,46^. Another factor that could either increase or decrease the binding of the PS to the bacterial envelope, leading to various photodynamic killing efficacies, is the capsular polysaccharide composition^47^. Additionally, as demonstrated by Tegos and Hamblin^48^, the possible mechanism determining aPDI efficacy could be efflux pumps, as several PSs may be substrates of these pumps. Other bacterial factors affecting phototreatment efficacy are the statuses of antioxidant enzymes and stress-induced *sigB* operons, as evidenced by Nakonieczna *et al*.^49,50^, the statuses of global transcriptional regulators (i.e., *agr*)^51,52^ and heat shock proteins^53^. The mentioned studies indicate possible factors that could affect bacterial susceptibility to photoinduced oxidative stress; thus, it could be assumed that if the genetic alterations accumulated upon sub-lethal aPDI/aBL treatment affect the activity and/or production of these factors, the mechanism underlying the observed tolerance development could be explained.

Additional significant finding described within the current manuscript is well-known phenomenon called photodynamic priming or hypersusceptibility that results in rendering microbes susceptible to various antimicrobials. This phenomenon has been deeply reviewed by Wozniak *et al*.^54^ together with our previously published data indicating aBL and aPDI to be synergistic with routinely used antibiotics^55^. The sequential use of sub-lethal aBL and or aPDI results in antibiotic MIC values reduction by 2-, and even 64-fold^55–58^. Although the photodynamic priming is a well-known phenomenon, the observed within the current study hypersusceptibility of aPDI/aBL tolerant isolate to doxycycline and gentamycin may not be termed simple “priming”. When considering synergy between phototreatment and antimicrobial, the pre-treatment of the microbe with sub-lethal photoinactivation followed with subsequent administration of an antibiotic results in increased susceptibility of the pre-treated cells and enhanced killing. This effect is limited and requires the pre-treatment of microbial cells with sub-lethal aPDI/aBL. It indicates that the “priming” effect is lost within culturing and is not transferred to next bacterial generation. Antimicrobial sensitization reported within the current manuscript is a stable phenotypic feature, transferred with culturing to next generations and expressing as reduced microbial resistance to described antibiotics, i.e., doxycycline and gentamycin. Along with reduced microbial drug resistance, the observed phenomenon supports the thesis of genetic alterations accumulated during several sub-lethal aPDI/aBL consecutive cycles that could constitute the developed tolerance.

In accordance with the SCCS report, the observed *S*. *aureus* aPDI/aBL tolerance is in line with the definition indicating the strain has reduced susceptibility to the antimicrobial treatments. Thus, the development of observed tolerance does not deny the successful application of phototreatments in the clinic but rather indicates the possible limitations that could be overcome with responsible treatment policies.

## Supplementary information

Supplementary Data 1

Supplementary Data 2

Supplementary Data 3

Supplementary Data 4

## Data Availability

The datasets generated during and/or analysed during the current study are available from the corresponding author on reasonable request.
